# A Variation of the Algorithm to Achieve the Maximum Entropy for Belief Functions

**DOI:** 10.3390/e25060867

**Published:** 2023-05-29

**Authors:** Joaquín Abellán, Alejandro Pérez-Lara, Serafín Moral-García

**Affiliations:** Department of Computer Science and Artificial Intelligence, University of Granada, 18014 Granada, Spain; alejandropl@correo.ugr.es (A.P.-L.); seramoral@decsai.ugr.es (S.M.-G.)

**Keywords:** belief functions, uncertainty measures, maximum of entropy

## Abstract

Evidence theory (TE), based on imprecise probabilities, is often more appropriate than the classical theory of probability (PT) to apply in situations with inaccurate or incomplete information. The quantification of the information that a piece of evidence involves is a key issue in TE. Shannon’s entropy is an excellent measure in the PT for such purposes, being easy to calculate and fulfilling a wide set of properties that make it axiomatically the best one in PT. In TE, a similar role is played by the maximum of entropy (ME), verifying a similar set of properties. The ME is the unique measure in TE that has such axiomatic behavior. The problem of the ME in TE is its complex computational calculus, which makes its use problematic in some situations. There exists only one algorithm for the calculus of the ME in TE with a high computational cost, and this problem has been the principal drawback found with this measure. In this work, a variation of the original algorithm is presented. It is shown that with this modification, a reduction in the necessary steps to attain the ME can be obtained because, in each step, the power set of possibilities is reduced with respect to the original algorithm, which is the key point of the complexity found. This solution can provide greater applicability of this measure.

## 1. Introduction

Managing uncertainty is essential for making decisions. Evidence theory (TE), also known as the Dempster–Shafer theory [1,2], is widely employed to handle uncertainty-based information in practical applications such as *medical diagnosis* [3], *statistical classification* [4], *target identification* [5], *face recognition* [6], or *risk management* [7,8]. TE is also commonly utilized to fuse information from different sources [9,10,11], a crucial issue for decision making.

Evidence theory extends classical Probability Theory (PT). It is based on the *basic probability assignment* concept (b.p.a.), a generalization of the concept of the probability distribution in PT. Each b.p.a. in TE has a belief function and a plausibility function associated with it. The belief (plausibility) value of a set is the minimum (maximum) support of information represented by the b.p.a. on that set.

In TE, it is essential to quantify the uncertainty-based information represented by a b.p.a. For this purpose, many uncertainty measures in TE have been proposed so far. The starting point of most of them is the Shannon entropy [12], a well-established uncertainty measure in PT that satisfies a large set of properties.

As TE generalizes PT, there are more types of uncertainty in TE than in PT. As pointed out by Yager [13], two types of uncertainty appear in TE. The first one, called *conflict*, arises when the information focuses on disjoint sets. The second type, known as *non-specificity*, appears when the information has a cardinality greater than or equal to two. Hence, a total uncertainty measure in TE must capture both the conflict and non-specificity.

Klir and Weirman [14] carried out a study concerning the set of mathematical properties that have to be verified by every uncertainty measure in TE. Such a study was extended by Abellán and Masegosa [15]. They also analyzed the behavioral requirements that a total uncertainty measure in TE must satisfy. The maximum entropy on the closed and convex set of probability distributions (credal set) compatible with a b.p.a., proposed in [16], is the only total uncertainty measure in TE so far that verifies all the crucial mathematical properties and behavioral requirements.

However, the algorithms proposed so far in [16,17] to compute the maximum entropy of the credal set associated with a b.p.a. are notably complex. For this reason, in recent years, many alternative measures to the maximum entropy have been introduced. Nonetheless, none of these measures verifies all the required mathematical properties and behaviors for uncertainty measures in TE [18,19,20]. This is the principal reason for the lack of consensus on the use of uncertainty measures in TE: the ME has an optimal axiomatic behavior but a high complexity of calculus, and more recent ones have had worse axiomatic behavior but a low complexity of calculus.

It must be remarked that the ME has been used with excellent results in practical applications, such as in the data mining area. Its use on special types of belief functions can be of different complexity, and its calculus is immediate. We can find examples of this in [21,22,23,24].

An approximation to the maximum entropy on the credal set associated with a b.p.a. was proposed in [25]. Such an approximation consisted of the maximum entropy on the credal set consistent with the belief intervals for singletons, where the lower and upper bounds were, respectively, the belief and plausibility values on singletons. Even though this measure satisfied all the crucial mathematical properties and behavioral requirements, when the belief intervals for singletons are employed to represent the uncertainty-based information instead of the corresponding b.p.a., some information could be lost because the credal set consistent with a b.p.a. is always contained in the one compatible with the associated belief intervals for singletons [25]. In consequence, this uncertainty measure indicated more uncertainty than the one represented by a b.p.a.

In this research, we propose a variation of the algorithm for computing the maximum entropy on the credal set compatible with a b.p.a. We demonstrate that our proposed procedure involves less computational time than the algorithms developed so far for the maximum entropy on the credal set corresponding to a b.p.a. With our proposal, fewer steps are necessary to achieve the probability distribution of maximum entropy on the credal set consistent with a b.p.a. This is shown via some numerical examples and with an experimentation over a huge set of b.p.a. functions randomly generated. The reduction in the computational time of our proposed algorithm makes this measure (maximum of entropy) more suitable for use in practical applications.

The remainder of this paper is structured as follows: Section 2 describes evidence theory, the main uncertainty measures proposed so far in evidence theory, and the algorithm developed so far to compute the maximum entropy on the credal set consistent with a basic probability assignment. Our proposed procedure and different examples of its use, compared with the original one, are presented in Section 3. Moreover, in that section, we present an experiment showing the gains in the time of computing obtained with the new improved algorithm compared with the original one. Concluding remarks and ideas for future work are given in Section 4.

## 2. Background

Let $X=\left\{x_{1},\ldots ,x_{t}\right\}$ be a finite set of possible alternatives, also known as the *frame of discernment*. Let ℘(X) denote the power set of *X*.

### 2.1. Theory of Evidence

Evidence theory (TE), also known as the Dempster–Shafer theory [1,2], is based on the concept of a *basic probability assignment* (b.p.a.). A b.p.a. is a mapping m:℘(X)→[0,1], satisfying m(∅)=0 and $\sum_{A\subseteq X}m\left(A\right)=1$.

If A⊆X verifies that m(A)>0, *A* is said to be a *focal element* of *m*.

A given b.p.a. *m* on *X* has a *belief function* $Bel_{m}$ and a *plausibility function* $Pl_{m}$ associated with it. Such functions are defined as follows:

(1) $$Bel_{m}\left(A\right)=\sum_{B|B\subseteq A}m\left(B\right),\;Pl_{m}\left(A\right)=\sum_{B|B\cap A\neq \emptyset }m\left(B\right),\;\forall A\subseteq X.$$

We may note that for each A⊆X, $Bel_{m}\left(A\right)\leq Pl_{m}\left(A\right)$. The interval $\left[Bel_{m}\left(A\right),Pl_{m}\left(A\right)\right]$ is called the *belief interval* of A∀A⊆X. In addition,

(2) $$Pl_{m}\left(A\right)=1-Bel_{m}\left(\bar{A}\right),\;\forall A\subseteq X,$$

where $\bar{A}$ denotes the complement of *A*. Thereby, $Bel_{m}$ and $Pl_{m}$ are called dual or conjugate. One of them is sufficient for representing the uncertainty-based information in TE. For this purpose, $Bel_{m}$ is often utilized.

For a given b.p.a. *m* on *X*, the set of probability distributions compatible with it (a closed and convex set of probability distributions, also called a credal set) is given by:

(3) $$P_{m}=\left\{p\in P\left(X\right)|Bel_{m}\left(A\right)\leq p\left(A\right)\;\forall A\subseteq X\right\},$$

where P(X) is the set of all probability distributions on *X*.

### 2.2. Uncertainty Measures in Evidence Theory

The Shannon entropy [12] is a well-established uncertainty measure in probability theory. For a probability distribution *p* on *X*, the Shannon entropy is defined as follows:

(4) $$S\left(p\right)=\sum_{i=1}^{t}-p\left(\left\{x_{i}\right\}\right)\log_{2}\left(p\left(\left\{x_{i}\right\}\right)\right).$$

The type of uncertainty measured by *S* is called *conflict*. It is the only type of conflict present in probability theory. It satisfies a set of desirable properties [12,14].

In classical possibility theory, the Hartley measure [26] is a well-established uncertainty measure. It is defined in the following way:

(5) $$H\left(A\right)=\log_{2}\left(\left|A\right|\right),\;\forall A\in ℘\left(X\right).$$

The type of uncertainty measured by *H*, the only one existing in possibility theory, is known as *non-specificity*.

As pointed out by Yager [13], conflict and non-specificity coexist in TE; conflict appears when the information is focused on disjoint sets, while non-specificity arises when the information resides in sets with a cardinality greater than one.

A generalization of the Hartley measure to TE was introduced by Dubois and Prade in [27]. It is given by:

(6) $$GH\left(m\right)=\sum_{A\subseteq X}m\left(A\right)\log_{2}\left(\left|A\right|\right).$$

GH reaches its minimum value, 0, when *m* is a probability distribution, and the maximum value of GH, $log_{2}\left(t\right)$, is attained when m(X)=1. GH is an appropriate non-specificity measure in TE that satisfies desirable properties. Moreover, it is easily extensible to more general theories than TE [28].

Several attempts to generalize the Shannon entropy to TE have been proposed, but none of them has satisfied all the essential requirements for this type of measure in TE: probability consistency, set consistency, range, additivity, subadditivity, and monotonicity [15].

Next, a total uncertainty measure in TE that quantified both the conflict and non-specificity was proposed. Such a measure, developed by Harmanec and Klir [16], is the maximum entropy on the credal set consistent with the b.p.a. *m*, $P_{m}$, determined via Equation (3). It is denoted by $S^{*}\left(P_{m}\right)$. This measure is suitable for quantifying uncertainty in TE because it is the only one so far that satisfies all necessary mathematical properties and behavioral requirements for uncertainty measures in TE.

Nevertheless, the algorithms proposed so far in [16,17] for computing $S^{*}\left(P_{m}\right)$ (also noted in the literature as $S^{*}\left(Bel\right)$ or $S^{*}\left(Bel_{m}\right)$, where all the expressions have the same meaning: the maximum of entropy over all the probability distributions associated with a b.p.a. *m*), are very complex. For this reason, in recent years, many alternative measures to $S^{*}\left(P_{m}\right)$ have been proposed.

For instance, the Deng entropy was presented in [18,29,30,31]. It was defined in the following way:

(7) $$E_{d}\left(m\right)=-\sum_{A\subseteq X}m\left(A\right)\log_{2}\left(\frac{m(A)}{2^{\left|A\right|}-1}\right)=\sum_{A\subseteq X}m\left(A\right)\log_{2}\left(2^{\left|A\right|}-1\right)-\sum_{A\subseteq X}m\left(A\right)\log_{2}\left(m(A)\right).$$

In Equation (7), the first term captures the non-specificity, while the second one quantifies the conflict part. The idea of this measure is that there must be more uncertainty as the number of alternatives increases. However, the Deng entropy violates most of the required mathematical properties for the uncertainty measures in TE, and its behavior in many cases is problematic [19].

The basis for some recent uncertainty measures in TE is the plausibility transformation [32,33], defined in the following way:

(8) $$Pt\left(x_{i}\right)=\frac{Pl_{m}\left(\left\{x_{i}\right\}\right)}{\sum_{j=1}^{t}Pl_{m}\left(\left\{x_{j}\right\}\right)},\;\forall i=1,\ldots ,t.$$

Jirousek and Shenoy [34] introduced a new uncertainty measure consisting of the sum of the GH and the Shannon entropy of the plausibility transformation:

(9) $$H_{JS}\left(m\right)=GH\left(m\right)-\sum_{i=1}^{t}Pt\left(x_{i}\right)\log_{2}\left(Pt(x_{i})\right).$$

The first term of Equation (9) captures the conflict, whereas the second one corresponds to the non-specificity.

In [35], an uncertainty measure also based on the plausibility transformation was proposed. It is defined as follows:

(10) $$H_{PQ}\left(m\right)=-\sum_{A\subseteq X}m\left(A\right)\log_{2}\left(Pm(A)\right)+GH\left(m\right),$$

where for each A⊆X, $m\left(A\right)=\sum_{x\in A}Pt\left(x\right)$. The first term quantifies the conflict, while the second one captures the non-specificity.

As shown in [25], the $H_{JS}$ does not satisfy all the required mathematical properties for the uncertainty measures in TE. The same situation occurs with the $H_{PQ}$.

Let us consider the set of belief intervals for singletons associated with *m*:

(11) $$I_{m}=\left\{\left[Bel_{m}\left(\left\{x_{i}\right\}\right),Pl_{m}\left(\left\{x_{i}\right\}\right)\right]\;i=1,\ldots ,t\right\}.$$

The uncertainty measure proposed in [36] combines the Deng entropy with the belief intervals for singletons. It is defined as:

(12) $$\begin{array}{cc} H_{inter}\left(m\right)= & -\sum_{x\in X}\frac{Bel_{m}\left(\left\{x\right\}\right)+Pl_{m}\left(\left\{x\right\}\right)}{2}\\ & \times \log_{2}\left[\frac{Bel_{m}\left(\left\{x\right\}\right)+Pl_{m}\left(\left\{x\right\}\right)}{2}\times \exp(-(Pl_{m}\left(\left\{x\right\}\right)-Bel_{m}\left(\left\{x\right\}\right)\right]\\ & -\sum_{A\subseteq X||A|\geq 2}m\left(A\right)\times \log_{2}\left[\frac{m(A)}{2^{\left|A\right|}-1}\exp\left(-\left(Pl_{m}\left(A\right)-Bel_{m}\left(A\right)\right)\right)\right]. \end{array}$$

In Equation (12), the first term captures the conflict, and the second one quantifies the non-specificity. As demonstrated in [25], the measure also does not satisfy all the crucial mathematical properties for the uncertainty measures in TE.

Let $P\left(I_{m}\right)$ denote the credal set consistent with the belief intervals for singletons associated with *m*. It is determined by:

(13) $$P\left(I_{m}\right)=\left\{p\in P\left(X\right)|Bel_{m}\left(\left\{x_{i}\right\}\right)\leq p\left(\left\{x_{i}\right\}\right)\leq Pl_{m}\left(\left\{x_{i}\right\}\right),\;\forall i=1,\ldots ,t\right\},$$

with P(X) being the set of all probability distributions on *X*.

In [25], an uncertainty measure that consists of the maximum entropy on the credal set given in Equation (13) was proposed:

(14) $$S^{*}\left(P\left(I_{m}\right)\right)=\max_{p\in P\left(I_{m}\right)}S\left(p\right).$$

$S^{*}\left(P\left(I_{m}\right)\right)$ verified all the essential mathematical properties and behavioral requirements for uncertainty measures in TE, as demonstrated in [25].

Nevertheless, it always holds that $P_{m}\subseteq P\left(I_{m}\right)$. Consequently, using $I_{m}$ rather than *m* to represent uncertainty may imply some loss of information. Therefore, $S^{*}\left(P\left(I_{m}\right)\right)$ might indicate more uncertainty than the one involved in *m*. The principal advantage of the use of the maximum entropy in this case is the notable reduction in the complexity though this implies a possible loss of information.

### 2.3. Algorithm to Compute the Maximum Entropy

For the calculation of the maximum entropy, it is necessary to solve a nonlinear optimization problem. To solve this issue, Meyerowitz et al. [17] proposed an algorithm for the calculation of the maximum entropy given a belief function. The algorithm follows these steps:

**Input:** A belief function *Bel* on the frame of discernment *X*.

1. Find a nonempty set A∈℘(X), such that $\frac{Bel(A)}{\left|A\right|}$ is maximal. If there exists more than one set *A* that attains that maximal, choose the one with maximal cardinality.
2. For x∈A, put $p_{x}=\frac{Bel(A)}{\left|A\right|}$.
3. For each B∈℘(X\A), put $Bel^{\prime }\left(B\right)=Bel\left(B\cup A\right)-Bel\left(A\right)$.
4. Put X=X\A.
5. If X≠∅ and $Bel^{\prime }\left(X\right)>0$, then go to Step 1.
6. If Bel(X)=0 and X≠∅, then put $p_{x}=0,\;\;\forall x\in X$.
7. Calculate $S^{*}\left(Bel\right)=-\sum_{x\in X}p_{x}\log_{2}p_{x}$.

Meyerowitz et al.’s algorithm provides a process for taking the probabilities that maximize the Shannon entropy, starting from a given belief function. This algorithm has a complexity of $2^{\left|X\right|}$ because in each iteration it is necessary to check which set maximizes $\frac{Bel(A)}{\left|A\right|},\;\forall A\subseteq X$.

## 3. A Computational Improvement of Meyerowitz et al.’s Algorithm

In the following, we present the improvement of Meyerowitz et al.’s algorithm. For this new procedure, we need to define an accumulator variable (acu), which we initialize by taking acu=1; this variable can be considered as the probability to be distributed among the elements of the frame of discernment X. The algorithm follows these steps:

**Input:** A belief function *Bel* on the frame of discernment *X*.

1. Find a nonempty set A∈℘(X), such that $\frac{Bel(A)}{\left|A\right|}$ is maximal. If there exists more than one set *A* that attains that maximal, choose the one with maximal cardinality.
2. Find a nonempty set B∈℘(X), such that $\frac{Bel(B)}{\left|B\right|}$ is minimal. If there exists more than one set *B* that attains that minimal, choose the one with minimal cardinality.
3. For x∈A, put $p_{x}=\frac{Bel(A)}{\left|A\right|}$.
4. For each y∈B, put $p_{y}=\frac{Pl(B)}{\left|B\right|}$.
5. Put acu=acu−Bel(A)−Pl(B).
6. For each $C\in ℘(X\backslash \left\{A\cup B\right\})$, put $Bel^{\prime }\left(C\right)=Bel\left(C\cup A\right)-Bel\left(A\right)$.
7. Put $X=X\backslash \left\{A\cup B\right\}$.
8. For each C∈℘(X), put $Pl^{\prime }\left(C\right)=acu-Bel^{\prime }\left(\bar{C}\right)$.
9. If X≠∅ and X≠∅, then go to the first step.
10. If Bel(X)=0 and X≠0, then put $p_{x}=0,\;\;\forall x\in X$.
11. Calculate $S^{*}\left(Bel\right)=-\sum_{x\in X}p_{x}\log_{2}p_{x}$.

Note that, for steps 1 and 2, the same set of subsets is used. We note this characteristic because it is important: we only need to calculate and use once the power set of *X*. We observe that the cardinality of the resulting frame of discernment is reduced more than in the original algorithm. We must remember that the use of the powerset of the frame of discernment is the principal drawback found in the original algorithm. Hence, if the size of such a set is reduced, it is obvious that we can gain time for the calculus of that measure.

### 3.1. Justification

The idea behind this algorithm is based on the use of the property $Pl\left(A\right)=1-Bel\left(\bar{A}\right)\;\forall A\in ℘\left(X\right)$, which relates the belief and plausibility functions. To achieve this, we take the original algorithm as a starting point. Let $A_{i}$(i∈{1,…,n}) be ordered disjoint sets, obtained after *n* iterations of Meyerowitz et al.’s algorithm. These sets are those that verify that $\frac{Bel(A)}{\left|A\right|}$ is maximal at each iteration of the algorithm (for simplicity, we assume that $p_{x}\neq 0:x\in A_{n}$. In the case where this is not satisfied, we consider the sets $A_{i}$ with 1≤i≤n−1).

Within the algorithm, we defined the function $Bel^{\prime }\left(B\right)=Bel\left(B\cup A_{1}\right)-Bel\left(A_{1}\right)$∀B⊆X\A. Now, our goal is to find a way to relate successive iterations of the function Bel to the function Pl. For this purpose, the following expression was proposed, where $Bel^{n}$ is the belief function at the n-th iteration of the original algorithm:

(15) $$Bel^{n}\left(A_{n+1}\right)=Bel\left(\cup_{i=1}^{n+1}A_{i}\right)-Bel\left(\cup_{i=1}^{n}A_{i}\right).$$

To proove the correctness of this equation, we give the following property.

**Proposition** **1.** *Let Bel and $Bel^{n}$ be the belief functions in the first and last iteration of Meyerowitz et al.’s algorithm, respectively. Then, the following is verified.*

(16) $$Bel^{n}\left(A_{n+1}\right)=Bel\left(\cup_{i=1}^{n+1}A_{i}\right)-Bel\left(\cup_{i=1}^{n}A_{i}\right).$$

**Proof.**  We use induction to prove the equality:

- For n=1:
  (17) $$Bel^{\prime }\left(A_{2}\right)=Bel\left(A_{2}\cup A_{1}\right)-Bel\left(A_{1}\right).$$
- For n=2:
  (18) $$\begin{array}{cc} Bel^{2}\left(A_{3}\right) & =Bel^{\prime }\left(A_{3}\cup A_{2}\right)-Bel^{\prime }\left(A_{2}\right),\\ Bel^{2}\left(A_{3}\right) & =Bel\left(A_{3}\cup A_{2}\cup A_{1}\right)-Bel\left(A_{1}\right)-Bel\left(A_{2}\cup A_{1}\right)+Bel\left(A_{1}\right),\\ Bel^{2}\left(A_{3}\right) & =Bel\left(A_{3}\cup A_{2}\cup A_{1}\right)-Bel\left(A_{2}\cup A_{1}\right). \end{array}$$
- Now, we assume true for *n*, then
  (19) $$\begin{array}{cc} Bel^{n+1}\left(A_{n+2}\right) & =Bel^{n}\left(A_{n+2}\cup A_{n+1}\right)-Bel^{n}\left(A_{n+1}\right)\\ \; & =Bel^{n}\left(A_{n+2}\cup A_{n+1}\right)-Bel\left(\cup_{i=1}^{n+1}A_{i}\right)+Bel\left(\cup_{i=1}^{n}A_{i}\right)\\ \; & =Bel^{n-1}\left(A_{n+2}\cup A_{n+1}\cup A_{n}\right)-Bel^{n-1}\left(A_{n}\right)-Bel\left(\cup_{i=1}^{n+1}A_{i}\right)+Bel\left(\cup_{i=1}^{n}A_{i}\right)\\ \; & =Bel^{n-1}\left(A_{n+2}\cup A_{n+1}\cup A_{n}\right)-Bel\left(\cup_{i=1}^{n}A_{i}\right)+Bel\left(\cup_{i=1}^{n-1}A_{i}\right)\\ \; & -Bel\left(\cup_{i=1}^{n+1}A_{i}\right)+Bel\left(\cup_{i=1}^{n}A_{i}\right)\\ \; & =Bel^{n-1}\left(A_{n+2}\cup A_{n+1}\cup A_{n}\right)+Bel\left(\cup_{i=1}^{n-1}A_{i}\right)-Bel\left(\cup_{i=1}^{n+1}A_{i}\right)\\ \; & \cdots \\ \; & =Bel^{\prime }\left(\cup_{i=2}^{n+2}A_{i}\right)+Bel\left(A_{1}\right)-Bel\left(\cup_{i=1}^{n+1}A_{i}\right)\\ \; & =Bel\left(\cup_{i=1}^{n+2}A_{i}\right)-Bel\left(A_{1}\right)+Bel\left(A_{1}\right)-Bel\left(\cup_{i=1}^{n+1}A_{i}\right)\\ \; & =Bel\left(\cup_{i=1}^{n+2}A_{i}\right)-Bel\left(\cup_{i=1}^{n+1}A_{i}\right). \end{array}$$

Hence, it is verified for n+1. □

Meyerowitz et al.’s algorithm ends after a finite number of steps; in our case, we will say n. Therefore, in the last iteration, we would obtain that $Bel^{n-1}\left(A_{n}\right)$ was the last set for which the probability was calculated. So, if we apply Equation (17), we obtain:

(20) $$\begin{array}{cc} Bel^{n-1}\left(A_{n}\right) & =Bel\left(\cup_{i=1}^{n}A_{i}\right)-Bel\left(\cup_{i^{1}}^{n-1}A_{i}\right)\\ \; & =1-Bel(\cup_{i^{1}}^{n-1}A_{i})\\ \; & =Pl(A_{n}). \end{array}$$

Having established this new relationship, we must take into account that, to implement it in the algorithm, it is necessary to take the minimum of the plausibility functions. This is because the relationship is between the original plausibility function and the belief function at the *n*-th iteration, which is the smallest of all.

The next step is to know how the successive plausibility functions are calculated over the iterations of the algorithm and how this affects the calculation of $Bel^{\prime }$. For this purpose, the following property is stated.

**Proposition** **2.** *The calculation of $Bel^{\prime }$ and $Pl^{\prime }$ follows the following equation:*

(21) $$Bel^{\prime }\left(C\right)=Bel\left(C\cup A_{1}\right)-Bel\left(A_{1}\right),\;\forall C\subseteq X\backslash \left\{A_{1}\cup A_{n}\right\}.$$

**Proof.**  We begin by studying what happens to the function $Bel^{\prime }$.

For the calculation of the function $Bel^{\prime }$, we do not take into account the set $A_{n}$; this was excluded and not taken into account in the following iterations.

We have to study how the function $Pl^{\prime }$ is calculated. For this purpose, we use Equation (15).

Considering the sets $A_{i}$ with 2≤i≤n−1, we have:

(22) $$Bel^{n-2}\left(A_{n-1}\right)=Bel^{\prime }\left(\cup_{i=2}^{n-1}A_{i}\right)-Bel^{\prime }\left(\cup_{i=2}^{n-2}A_{i}\right).$$

In the improvement of the algorithm, we defined $Bel^{\prime }\left(\cup_{i=2}^{n-1}A_{i}\right)=acu$. Hence, making use of (15), we have:

(23) $$Bel^{n-2}\left(A_{n-1}\right)=acu-Bel^{\prime }\left(\cup_{i=2}^{n-2}A_{i}\right).$$

In addition, since the set $A_{i}:\;i\in \left\{1,\ldots ,n\right\}$ is disjoint, and we defined the new frame of discernment as $X\backslash \left\{A_{1}\cup A_{n}\right\}$, we have that the set $\cup_{i=2}^{n-2}A_{i}=\bar{A_{n-1}}$. Therefore, we obtain:

(24) $$Pl^{\prime }\left(A_{n-1}\right)=acu-Bel^{\prime }\left(\cup_{i=2}^{n-2}A_{i}\right).$$

□

### 3.2. Example 1

Given the frame of discernment X={a,b,c,d}, if we take the belief function Bel defined by the following basic probability assignment *m* (see [14]), then:

$$\begin{array}{cc} m(\{a\}) & =0.26\\ m(\{b\}) & =0.26\\ m(\{c\}) & =0.26\\ m(\{a,b\}) & =0.07\\ m(\{a,c\}) & =0.01\\ m(\{a,d\}) & =0.01\\ m(\{b,c\}) & =0.01\\ m(\{b,d\}) & =0.01\\ m(\{c,d\}) & =0.01\\ m(\{a,b,c,d\}) & =0.1 \end{array}$$

- **Meyerowitz et al.’s algorithm**
  - **First iteration:** The maximum of the function $\frac{Bel(A)}{\left|A\right|}\;\forall A\subseteq X$ is reached for A={a,b}. So, we have $p_{a}=p_{b}=0.295$. Now, we update the function Bel, and as X={c,d}, and there are sets whose function Bel is nonzero, we need a second iteration.
  - **Second iteration:** The maximum of the function $\frac{Bel(A)}{\left|A\right|}\;\forall A\subseteq X$ is attained for A={c}. Thereby, it holds that $p_{c}=0.28$. Now, we update the function Bel, and since X={d}, and there are sets whose function Bel is nonzero, we need a third iteration.
  - **Third iteration:** For this last iteration, we have that X={d}, and we have that $Bel\left(\left\{d\right\}\right)=p_{d}=0.13$. With this, we arrive at X=∅; so, we can now calculate $S^{*}\left(Bel\right)$.
    Now, we proceed to the calculation of the maximum entropy:
    (25) $$\begin{array}{cc} S^{*}\left(Bel\right)= & -\sum_{i\in \{a,b,c,d\}}p_{i}\log_{2}p_{i}\\ = & -2\times 0.295\log_{2}\left(0.295\right)-0.28\log_{2}\left(0.28\right)-0.13\log_{2}\left(0.13\right)\\ = & 1.93598. \end{array}$$
- **Improvement of Meyerowitz et al.’s algorithm**
  - **First iteration:** The maximum of the function $\frac{Bel(A)}{\left|A\right|}\;\forall A\subseteq X$ is reached for A={a,b}. Therefore, we have $p_{a}=p_{b}=0.295$. Now, we see the minimum value of $\frac{Pl(B)}{\left|B\right|}\;\forall B\subseteq X$, which in this case is B={d}, and we have $p_{d}=0.13$. We update the value of acu=0.28, the function Bel, and the function Pl, and since X={c}, and there are sets whose function Bel is nonzero, we need a second iteration.
  - **Second iteration:** The maximum of the function $\frac{Bel(A)}{\left|A\right|}\;\forall A\subseteq X$ is attained for A={c}. So, it is satisfied that $p_{c}=0.28$. Now, we see the minimum value of $\frac{Pl(B)}{\left|B\right|}\;\forall B\subseteq X$, which in this case is the same that we obtained for *A*, B={c}, and we have $p_{c}=0.28$. With this we arrive at X=∅. Now, we can calculate $S^{*}\left(Bel\right)$.
    Now, we proceed to the calculation of the maximum entropy:
    (26) $$\begin{array}{cc} S^{*}\left(Bel\right)= & -\sum_{i\in \{a,b,c,d\}}p_{i}\log_{2}p_{i}\\ = & -2\times 0.295\log_{2}\left(0.295\right)-0.28\log_{2}\left(0.28\right)-0.13\log_{2}\left(0.13\right)\\ = & 1.93598. \end{array}$$

In this example, we reduced one step, i.e., we needed the calculus of the power set one time less. In this case, we can observe that the probability distribution where the maximum of entropy was attained, was very close to the initial mass values of the singletons. Hence, the original algorithm needed little effort to find that maximum. Thus, the improvement with the new algorithm was not large.

### 3.3. Example 2

Given the frame of discernment X={a,b,c,d,e,f}, we take the belief function Bel defined by the following basic probability assignment *m*:

$$\begin{array}{cc} m(\{a\}) & =0.25,\\ m(\{a,b\}) & =0.18,\\ m(\{a,b,c\}) & =0.16,\\ m(\{a,b,c,d\}) & =0.15,\\ m(\{a,b,c,d,e\}) & =0.14,\\ m(\{a,b,c,d,e,f\}) & =0.12. \end{array}$$

- **Meyerowitz et al.’s algorithm**
  - **First iteration:** The maximum of the function $\frac{Bel(A)}{\left|A\right|}\;\forall A\subseteq X$ is reached for A={a}. So, we have $p_{a}=0.25$. Now, we update the function Bel, and as X={b,c,d,e,f}, and there are sets whose function Bel is nonzero, we need a second iteration.
  - **Second iteration:** The maximum of the function $\frac{Bel(A)}{\left|A\right|}\;\forall A\subseteq X$ is attained for A={b}. Thereby, it holds that $p_{b}=0.18$. Now, we update the function Bel, and since X={c,d,e,f}, and there are sets whose function Bel is nonzero, we need a third iteration.
  - **Third iteration:** The maximum of the function $\frac{Bel(A)}{\left|A\right|}\;\forall A\subseteq X$ is reached for A={c}. In this way, we have $p_{c}=0.16$. Now, we update the function Bel, and as X={d,e,f}, and there are sets whose function Bel is non-zero, we need a fourth iteration.
  - **Fourth iteration:** The maximum of the function $\frac{Bel(A)}{\left|A\right|}\;\forall A\subseteq X$ is attained for A={d}. Hence, it is satisfied that $p_{d}=0.15$. Now, we update the function Bel, and since X={e,f}, and there are sets whose function Bel is nonzero, we need a fifth iteration.
  - **Fifth iteration:** The maximum of the function $\frac{Bel(A)}{\left|A\right|}\;\forall A\subseteq X$ is reached for A={e}. Consequently, it holds that $p_{e}=0.14$. Now, we update the function Bel, and as X={f}, and there are sets whose function Bel is nonzero, we need a sixth iteration.
  - **Sixth iteration:** For this last iteration, we have X={f}, and we have $Bel\left(\left\{f\right\}\right)=p_{f}=0.12$. With this, we arrive at X=∅; so, we can now calculate $S^{*}\left(Bel\right)$.
    Now, we proceed to the calculation of the maximum entropy:
    (27) $$\begin{array}{cc} S^{*}\left(Bel\right)= & -\sum_{i\in \{a,b,c,d,e,f\}}p_{i}\log_{2}p_{i}\\ = & -0.25\log_{2}\left(0.25\right)-0.18\log_{2}\left(0.18\right)-0.16\log_{2}\left(0.16\right)\\ \; & -0.15\log_{2}\left(0.15\right)-0.14\log_{2}\left(0.14\right)-0.12\log_{2}\left(0.12\right)\\ = & 2.5430. \end{array}$$
- **Improvement of Meyerowitz et al.’s algorithm**
  - **First iteration:** The maximum of the function $\frac{Bel(A)}{\left|A\right|}\;\forall A\subseteq X$ is reached for A={a}. Therefore, we have $p_{a}=0.25$. Now, we see the minimum value of $\frac{Pl(B)}{\left|B\right|}\;\forall B\subseteq X$, which in this case is B={f}, and we have that $p_{f}=0.12$. We update the value of acu=0.63, the function Bel, and the function Pl, and since X={b,c,d,e}, and there are sets whose function Bel is nonzero, we need a second iteration.
  - **Second iteration:** The maximum of the function $\frac{Bel(A)}{\left|A\right|}\;\forall A\subseteq X$ is attained for A={b}. So, it is satisfied that $p_{b}=0.18$. Now, we see the minimum value of $\frac{Pl(B)}{\left|B\right|}\;\forall B\subseteq X$, which in this case is B={e}, and we have that $p_{e}=0.14$. We update the value of acu=0.31, the function Bel, and the function Pl, and as X={c,d}, and there are sets whose function Bel is nonzero, we need a third iteration.
  - **Third iteration:** The maximum of the function $\frac{Bel(A)}{\left|A\right|}\;\forall A\subseteq X$ is reached for A={c}. Thus, we have $p_{c}=0.16$. Now, we see the minimum value of $\frac{Pl(B)}{\left|B\right|}\;\forall B\subseteq X$, which in this case is B={d}, and we have that $p_{d}=0.15$. With this, we arrive at X=∅. Now, we can calculate $S^{*}\left(Bel\right)$.
    Now, we proceed to the calculation of the maximum entropy:
    (28) $$\begin{array}{cc} S^{*}\left(Bel\right)= & -\sum_{i\in \{a,b,c,d,e,f\}}p_{i}\log_{2}p_{i}\\ = & -0.25\log_{2}\left(0.25\right)-0.18\log_{2}\left(0.18\right)-0.16\log_{2}\left(0.16\right)\\ \; & -0.15\log_{2}\left(0.15\right)-0.14\log_{2}\left(0.14\right)-0.12\log_{2}\left(0.12\right)\\ = & 2.5430. \end{array}$$

In this example, we reduced the number of steps to the middle one. This example used a b.p.a. with no conflict because all the focal sets shared an element.

### 3.4. Example 3

Given the frame of discernment X={a,b,c,d,e,f,g,h}, we take the belief function Bel defined by the following basic probability assignment *m*:

$$\begin{array}{cc} m(\{a\}) & =0.23,\\ m(\{g\}) & =0.05,\\ m(\{a,b\}) & =0.21,\\ m(\{g,h\}) & =0.02,\\ m(\{a,b,c\}) & =0.18,\\ m(\{a,b,c,d\}) & =0.13,\\ m(\{a,b,c,d,e\}) & =0.1,\\ m(\{a,b,c,d,e,f\}) & =0.08. \end{array}$$

- **Meyerowitz et al.’s algorithm**
  - **First iteration:** The maximum of the function $\frac{Bel(A)}{\left|A\right|}\;\forall A\subseteq X$ is reached for A={a}. So, we have $p_{a}=0.23$. Now, we update the function Bel, and as X={b,c,d,e,f,g,h}, and there are sets whose function Bel is nonzero, we need a second iteration.
  - **Second iteration:** The maximum of the function $\frac{Bel(A)}{\left|A\right|}\;\forall A\subseteq X$ is attained for A={b}. Thereby, it holds that $p_{b}=0.21$. Now, we update the function Bel, and since X={c,d,e,f,g,h}, and there are sets whose function Bel is nonzero, we need a third iteration.
  - **Third iteration:** The maximum of the function $\frac{Bel(A)}{\left|A\right|}\;\forall A\subseteq X$ is reached for A={c}. In this way, we have $p_{c}=0.18$. Now, we update the function Bel, and as X={d,e,f,g,h}, and there are sets whose function Bel is nonzero, we need a fourth iteration.
  - **Fourth iteration:** The maximum of the function $\frac{Bel(A)}{\left|A\right|}\;\forall A\subseteq X$ is attained for A={d}. Hence, it is satisfied that $p_{d}=0.13$. Now, we update the function Bel, and since X={e,f,g,h}, and there are sets whose function Bel is nonzero, we need a fifth iteration.
  - **Fifth iteration:** The maximum of the function $\frac{Bel(A)}{\left|A\right|}\;\forall A\subseteq X$ is reached for A={e}. Consequently, it holds that $p_{e}=0.1$. Now, we update the function Bel, and as X={f,g,h}, and there are sets whose function Bel is nonzero, we need a sixth iteration.
  - **Sixth iteration:** The maximum of the function $\frac{Bel(A)}{\left|A\right|}\;\forall A\subseteq X$ is reached for A={f}. In this way, we have $p_{f}=0.08$. Now, we update the function Bel, and as X={g,h}, and there are sets whose function Bel is nonzero, we need a seventh iteration.
  - **Seventh iteration:** The maximum of the function $\frac{Bel(A)}{\left|A\right|}\;\forall A\subseteq X$ is reached for A={g}. Hence, it is satisfied that $p_{g}=0.05$. Now, we update the function Bel, and as X={h}, and there are sets whose function Bel is nonzero, we need a eighth iteration.
  - **Eighth iteration:**
    For this last iteration, we have X={h}, and we have $Bel\left(\left\{h\right\}\right)=p_{h}=0.02$. With this, we arrive at X=∅; so, we can now calculate $S^{*}\left(Bel\right)$.
    Now, we proceed to the calculation of the maximum entropy:
    (29) $$\begin{array}{cc} S^{*}\left(Bel\right)= & -\sum_{i\in X}p_{i}\log_{2}p_{i}\\ = & -0.23\log_{2}\left(0.23\right)-0.21\log_{2}\left(0.21\right)-0.18\log_{2}\left(0.18\right)\\ \; & -0.13\log_{2}\left(0.13\right)-0.1\log_{2}\left(0.1\right)-0.08\log_{2}\left(0.08\right)\\ \; & -0.05\log_{2}\left(0.05\right)-0.02\log_{2}\left(0.02\right)\\ = & 2.74112. \end{array}$$
- **Improvement of Meyerowitz et al.’s algorithm**
  - **First iteration:** The maximum of the function $\frac{Bel(A)}{\left|A\right|}\;\forall A\subseteq X$ is reached for A={a}. Therefore, we have $p_{a}=0.23$. Now, we see the minimum value of $\frac{Pl(B)}{\left|B\right|}\;\forall B\subseteq X$, which in this case is B={h}, and we have $p_{h}=0.02$. We update the value of acu=0.75, the function Bel, and the function Pl, and since X={b,c,d,e,f,g}, and there are sets whose function Bel is nonzero, we need a second iteration.
  - **Second iteration:** The maximum of the function $\frac{Bel(A)}{\left|A\right|}\;\forall A\subseteq X$ is attained for A={b}. So, it is satisfied that $p_{b}=0.21$. Now, we see the minimum value of $\frac{Pl(B)}{\left|B\right|}\;\forall B\subseteq X$, which in this case is B={g}, and we have that $p_{g}=0.05$. We update the value of acu=0.49, the function Bel, and the function Pl, and as X={c,d,e,f}, and there are sets whose function Bel is nonzero, we need a third iteration.
  - **Third iteration:** The maximum of the function $\frac{Bel(A)}{\left|A\right|}\;\forall A\subseteq X$ is reached for A={c}. Thus, we have $p_{c}=0.18$. Now, we see the minimum value of $\frac{Pl(B)}{\left|B\right|}\;\forall B\subseteq X$, which in this case is B={f}, and we have that $p_{f}=0.08$. We update the value of acu=0.23, the function Bel, and the function Pl, and as X={d,e}, and there are sets whose function Bel is nonzero, we need a fourth iteration.
  - **Fourth iteration:** The maximum of the function $\frac{Bel(A)}{\left|A\right|}\;\forall A\subseteq X$ is reached for A={d}. Thus, we have $p_{d}=0.13$. Now, we see the minimum value of $\frac{Pl(B)}{\left|B\right|}\;\forall B\subseteq X$, which in this case is B={e}, and we have $p_{e}=0.1$. With this we arrive at X=∅. Consequently, we can now calculate $S^{*}\left(Bel\right)$.
    Now, we proceed to the calculation of the maximum entropy:
    (30) $$\begin{array}{cc} S^{*}\left(Bel\right)= & -\sum_{i\in X}p_{i}\log_{2}p_{i}\\ = & -0.23\log_{2}\left(0.23\right)-0.21\log_{2}\left(0.21\right)-0.18\log_{2}\left(0.18\right)\\ \; & -0.13\log_{2}\left(0.13\right)-0.1\log_{2}\left(0.1\right)-0.08\log_{2}\left(0.08\right)\\ \; & -0.05\log_{2}\left(0.05\right)-0.02\log_{2}\left(0.02\right)\\ = & 2.74112. \end{array}$$

Again, in this example we needed the middle steps to find the maximum of entropy. In this case, we used a b.p.a. with conflict. Clearly, the greater the |X|, the greater the improvement.

### 3.5. Experiments

We carried out a series of experiments generating b.p.a.s on sets of n=4, n=5, and n=6 elements (sizes of the frames of discernment), to examine the time of processing of both algorithms: the original and the improved ones. We implemented both algorithms in C++ programming language and ran them in a computer with an Intel Core i5 processor, a CPU of 1.8 GHz, and 8 G of RAM. Each b.p.a. was randomly generated with assigned mass values with the constraint that none of them was above 0.5/n. This characteristic ensured we could obtain b.p.a.s with a good level of sharing. Obviously, if a b.p.a. has masses only on the singlenton sets or only it is focused on one set, both algorithms are inmediate and similar, but these situations do not appear with the constraint used. We think that, with this constraint, the differences of both algorithms can be seen in a better way. The results can be seen in Table 1.

As we can see in Table 1, the reduction in time increases with the number of values. We had about an 8% improvement on sets of four elements, more than a 15% improvement on sets of five elements, and a more than 19% improvement on sets of six elements. As we showed in the previous examples, the level of improvement increased with the number of elements. The increment in the performance was duplicated from n=4 to n=6, with n=6 very close to 20%, which shows the importance of the new algorithm when the size of the frame of discernment increases.

As expected, we observed that the time of processing increased strongly with the size of the frame of discernment, which is a characteristic of algorithms that need to enumerate all the elements of the powerset of a universal set. However, we should not forget that the number of the first two columns correspond to seconds taken for the application of the algorithms on 10 million b.p.a.s on each set of size *n*. Hence, the average time of the calculus for obtaining the maximum entropy for one b.p.a. can be achieved dividing those values (from columns 1 and 2) by 10 million.

## 4. Conclusions and Future Work

The computational cost of Meyerowitz et al.’s algorithm has been the principal drawback of using the maximum entropy as a measure to quantify the uncertainty-based information in TE. In this work, a variation of that algorithm was presented. The key point of this new proposal is the double use of each enumeration of the subsets of the power set of the frame of discernment. This new procedure implies an important reduction in the set of elements in the resulting frame of discernment of each step in Meyerowitz et al.’s algorithm. Hence, the number of steps necessary to achieve the maximum entropy of a b.p.a. is reduced. The experiments carried out showed that the improvement in the time can be close to 20% for a set with a cardinality of six elements, showing that this improvement can be greater when we increase the size of the frame of discernment. The outcome presented may have greater applicability in TE. As future work and application of the algorithm presented here, we want to apply it in some real areas, such as the one based on the information obtained by sensors, where TE has been recently widely used.

We believe that it is possible to continue lowering the complexity of the calculation of the maximum entropy via new algorithms. Obtaining these new algorithms with better computational behavior and their comparison with those used in this work are part of our future work in this area of research.

## Figures and Tables

**Table 1:** Time of processing (seconds) of the calculus of both algorithms on 10 million b.p.a.s randomly generated on sets of 4, 5, and 6 elements.

	Original Alg.	Improved Alg.	Percentage of Improvement
n=4	29.10	26.69	8.28%
n=5	120.05	101.20	15.70%
n=6	1077.24	868.66	19.36%

## Data Availability

Not applicable.
